# A New Model for Bone Health Management in Postmenopausal Early Breast Cancer Patients Undergoing Adjuvant Endocrine Therapy—The Predict & Prevent Project

**DOI:** 10.3390/healthcare13243292

**Published:** 2025-12-15

**Authors:** Stefania Gori, Alessandra Fabi, Rossana Berardi, Paola Villa, Alberto Zaniboni, Tiziana Prochilo, Claudia Bighin, Alessandro Del Conte, Ferdinando Riccardi, Mario Airoldi, Alessandra Chirco, Saverio Cinieri, Armando Orlandi, Martina Assanti, Matteo Valerio, Roberto Tessari, Carlotta Mantoan, Matteo Verzè, Fabio Puglisi, Fabrizio Nicolis

**Affiliations:** 1 Medical Oncology, IRCCS Sacro Cuore Don Calabria Hospital, 37024 Negrar di Valpolicella, Verona, Italy; stefania.gori@sacrocuore.it; 2 Precision Medicine Unit in Senology, Fondazione Policlinico Universitario Agostino Gemelli, 00168 Rome, Italy; alessandra.fabi@policlinicogemelli.it (A.F.);; 3 Department of Oncology, Università Politecnica delle Marche, Azienda Ospedaliera Universitaria delle Marche, 60124 Ancona, Italy; r.berardi@staff.univpm.it; 4 Department of Women’s and Child Health and Public Health Science, Fondazione Policlinico Universitario Agostino Gemelli IRCCS, 00168 Rome, Italy; 5 Department of Clinical Oncology, Fondazione Poliambulanza Istituto Ospedaliero, 25124 Brescia, Italy; 6 Oncologia Medica 2, Ospedale Policlinico San Martino—IRCCS, 16132 Genoa, Italy; 7 SOC Oncologia Medica e dei Tumori Immunocorrelati, Centro di Riferimento Oncologico di Aviano (CRO) IRCCS, 33081 Aviano, Italy; 8 UOC Oncologia AORN Cardarelli Napoli, 80131 Naples, Italy; 9 SC Oncologia Medica 2, A.O.U. Città della Salute e della Scienza di Torino, 10126 Turin, Italy; 10 ASST Papa Giovanni XXIII—SC Oncologia, 24127 Bergamo, Italy; achirco@asst-pg23.it; 11 Medical Oncology, Antonio Perrino Hospital, 72100 Brindisi, Italy; 12 IQVIA Solutions Italy S.r.l, 20124 Milano, Italy; 13 Hospital Pharmacy, IRCCS Sacro Cuore Don Calabria Hospital, 37024 Negrar di Valpolicella, Verona, Italy; 14 Nurse Direction Unit of Clinical Cancer Center, IRCCS Sacro Cuore Don Calabria Hospital, 37024 Negrar di Valpolicella, Verona, Italy; carlotta.mantoan@aulss9.veneto.it; 15 Health Professions Service, Ospedale Fracastoro—San Bonifacio, Azienda ULSS9 Scaligera, 37122 Verona, Italy; 16 Medical Direction, IRCCS Sacro Cuore Don Calabria Hospital, 37024 Negrar di Valpolicella, Verona, Italy; 17 Department of Medical Oncology, Centro di Riferimento Oncologico (CRO) IRCCS, 33081 Aviano, Italy; fabio.puglisi@cro.it; 18 Department of Medicine, University of Udine, 33100 Udine, Italy

**Keywords:** hormonal receptor-positive early breast cancer, adjuvant endocrine therapy, bone health management model

## Abstract

**Objective:** The Italian Drug Agency (AIFA) Determination n. 589 of 2015 (Note 79) establishes that the use of bisphosphonates or denosumab is necessary for the primary prevention of bone fractures in postmenopausal patients with early breast cancer (EBC) undergoing adjuvant endocrine therapy (ET). Since adherence to the 2015 AIFA recommendation was still very low in 2019, a new bone health management model was identified to improve adherence to this recommendation. **Methods:** The aim of this project (Predict & Prevent) was to increase the percentage of patients with early breast cancer (EBC) with hormone receptor-positive (HR+) tumors treated. The project identified a new bone health model of management including the following: training of breast multidisciplinary teams and bone health specialists; presentation and implementation of this model in cancer centers; evaluation, at baseline and 12 months after the implementation of the project, of two key performance indicators (KPIs): rate of HR+ EBC patients assessed for bone health within 30 days from the start of adjuvant ET (KPI-1) and rate of HR+ breast cancer patients receiving bisphosphonates or denosumab within 90 days from the start of adjuvant ET (KPI-2). The primary endpoints of this study were the assessment of the rates of the two key performance indicators (KPIs) 12 months after the start of the project (T3) in comparison with the rates recorded at time 0 (T0) in each participating cancer center and the bone fracture rates at 5 years. In this first analysis, we reported the rates of two KPIs 12 months after the start of the new model (T3) and the comparison with the rates recorded at time 0 (T0) in each participating cancer center, to assess whether these percentages had increased after the implementation of the new organizational model. The rates of bone fractures will be evaluated after five years from implementation of this project in every cancer center. **Results:** From 2020 to 2022, 10 Italian cancer centers were involved in this project. As of September 2023, 9 cancer centers reported rates relative to two KPIs assessed in each hospital. In 6 hospitals (Negrar, Brescia, Bergamo, Aviano, Turin, Rome), the rates relative to KPI-1 and to KPI-2 increased progressively from time T0 (at baseline) to time T3 (after 12 months from the start of the project), due to training of multidisciplinary teams and implementation of a new bone health management model. In the other three cancer centers (Ancona, Genoa, Naples), where the rate of evaluation of bone health (KPI-1) and the indication for bisphosphonates/denosumab (KPI-2) in HR+ EBC patients were already high at time T0, the rates remained high even after 12 months from the start of this project. **Conclusions:** After 12 months from the implementation of this new organizational model of bone health management, an increase in the rate of postmenopausal HR+ EBC patients on adjuvant ET assessed for bone health and the rate of patients treated with bisphosphonate/denosumab were reported in six out of nine cancer centers. In the other three cancer centers, where the rates were high at baseline, the rates also remained high after 12 months from the new model implementation. This new model should be adopted in all cancer centers to allow adequate management of bone health in all postmenopausal HR+ EBC patients undergoing adjuvant ET, with the ultimate goal of reducing the rate of bone fractures in these patients in subsequent years.

## 1. Introduction

Worldwide, breast cancer is the most commonly diagnosed cancer (24.5% of 9.2 million new cases diagnosed in 2020) and the leading cause of tumor death in women (15.5% of 4.4 million deaths for cancer) [1]. Breast cancer represents the most frequently diagnosed neoplasm also in Europe [2] and in Italy [3] (355,457 and 54,976 new cases in 2020, respectively) and the first cause of oncological death in women (91,826 deaths in Europe and 12,995 in Italy) [2,4]. Due to the high incidence and good prognosis (5-year survival of 81.8% in Europe [5] and 87% in Italy [4]), the prevalence is very high. In 2020, there were 12.2 million women alive with a previous diagnosis of breast cancer in Europe [5] and 834,154 in Italy [3] and it is very important to protect the Quality of Life of these women during and after antitumoral treatments.

About 90–95% of breast cancers are diagnosed at an early stage (only 5–10% of new breast cancers are de novo metastatic), and about 80% of these cancers are hormone receptor-positive (HR+). The integration of the various therapeutic modalities (surgery, radiotherapy, (neo)-adjuvant systemic therapy) and the collaboration of various specialists within multidisciplinary teams are fundamental to ensure the best treatment for each patient and achieve the best outcome [3,4].

Adjuvant endocrine therapy (ET) is recommended in HR+ early breast cancer (EBC) by international and national guidelines [6,7] based on evidence of statistically and clinically significant reduction in recurrence and mortality from breast cancer [8,9,10].

Tamoxifen, a selective estrogen receptor modulator (SERM with tissue-specific estrogen receptor agonist/antagonist activity) and aromatase inhibitors (AIs—anastrozole, letrozole, exemestane—which inhibit estrogen synthesis) with or without ovarian function suppression (based on premenopausal or postmenopausal status at diagnosis) are still the most important adjuvant endocrine therapies. Ovarian function suppression (OFS) can be achieved by monthly administration of gonadotropin-releasing hormone (GnRH) agonists, bilateral oophorectomy, or bilateral ovarian irradiation [11].

Adjuvant ET has a duration of at least five years and, although it is generally well tolerated, it can cause side effects such as joint pain, osteopenia, osteoporosis, and bone fragility. Indeed, the marked tissue hypoestrogenism induced by adjuvant ET leads to accelerated bone loss and impaired bone structure quality, resulting in an increased risk of fractures, which often occur even with normal bone density. Evidence from clinical trials reported that in premenopausal patients, each adjuvant ET (tamoxifen, tamoxifen +OFS, AI+OFS) increases bone loss, with a bone mineral density (BMD) decline (2% per year with tamoxifen and 11% with AI+OFS [12,13,14,15,16]) and an increased rate of fractures (7.7% with AIs+OFS, 6% with tamoxifen+OFS and 5.3% with tamoxifen alone) [10]. In postmenopausal patients adjuvant therapy with AIs is associated with greater bone turnover, bone loss, and also fracture risk in comparison to tamoxifen [9,17,18,19,20,21].

However, in a clinical setting, two thirds of fractures are asymptomatic and a real-world study reported morphometric vertebral fractures in 31.2% of aromatase inhibitor-treated patients in comparison with 18.9% of aromatase inhibitor-naïve patients (*p* < 0.05) [22].

Cancer treatment-induced bone loss (CTIBL) and the subsequent increased risk of fragility fractures are clinically relevant concerns, with a significant impact on morbidity and quality of life [12,23,24] and an evaluation of other risk factors for osteoporosis and assessment of BMD with dual-energy X-ray absorptiometry (DXA) scanning at baseline and during adjuvant ET are also recommended [24] by ESMO guidelines.

Some clinical trials showed that the use of bisphosphonates or denosumab (which inhibit bone resorption through different mechanisms of action) during adjuvant endocrine therapy increases or preserves BMD and reduces the risk of fractures [15,25,26,27,28,29,30,31]. Zoledronic acid is the only bone-targeted drug effective in preventing BMD loss in randomized studies in premenopausal [15,25,26] and in postmenopausal patients [27,28,29], but with limited evidence of a reduction in fracture risk (not evaluated or evaluated as a secondary end point in clinical studies). Nevertheless, the EBCTCG meta-analysis, including 18,766 HR+/HR-negative pre- and post-menopausal EBC patients receiving bisphosphonates or placebo, reported information on fracture as secondary endpoint in 13,341 patients: a lower rate of fracture was observed in the bisphosphonate arm (6.3%) versus the control arm (7.3%) (RR 0.85; 95% CI 0.75–0.97; *p* = 0.02) [30].

Denosumab is the only bone-targeted agent which demonstrated prevention of fractures in HR+ EBC postmenopausal patients receiving AIs in the randomized phase 3 trial ABCSG-18. This study evaluated the time to first clinical fracture as the primary end point. After randomization of 3425 patients to receive denosumab (60 mg subcutaneously every 6 months for 5 years with calcium and vitamin D supplementation) or placebo, at a median follow up of 39 months, a significantly delayed time to first clinical fracture with denosumab compared with the placebo was reported (HR = 0.50; 95% CI 0.39–0.65; *p* < 0.0001). The overall lower number of fractures in the denosumab group (92) versus the placebo group (176) was similar in all patient subgroups, independent of age and basal bone mineral density (BMD) [31]. These results were confirmed at 11 years after randomization, with clinical fractures in 15.9% of denosumab patients compared with 19.2% in the placebo group.

To date, there are no data on a direct comparison between denosumab and bisphosphonates for primary fracture prevention in postmenopausal patients receiving adjuvant ET.

Based on this evidence, the Italian guidelines from AIOM [7,32,33] recommended in postmenopausal (natural, surgical, from chemotherapy or GnRH) HR+ EBC patients receiving adjuvant endocrine therapy, bisphosphonates, and denosumab for primary prevention of bone fractures.

In 2015, the Italian Drug Agency (AIFA—Agenzia Italiana del Farmaco) published Determination no. 589, known as “Note 79,” stating that the use of zoledronic acid (5 mg intravenously every 12 months), alendronate (70 mg orally per week ± vitamin D), risedronate (35 mg orally per week), and denosumab (60 mg subcutaneously every 6 months) is indicated for the primary prevention of bone fractures in women with postmenopausal breast cancer undergoing adjuvant ET, regardless of T-score values [34]. This treatment was approved and covered by the National Health Service (NHS). This authorization applies at the same dosages recommended for osteoporotic women. The intake or supplementation of vitamin D and calcium in the diet is also recommended.

However, a previous national survey conducted by IQVIA (a global healthcare data and clinical research company) in 2019 in 30 Italian cancer centers with identification of 1996 EBC patients with an indication for adjuvant ET, showed poor implementation of AIFA indications: only 25% of patients received bisphosphonate or denosumab therapy within 90 days of starting adjuvant ET (unpublished data).

Since adherence to the 2015 AIFA recommendation was still very low in 2019, a new bone health management model was identified to improve adherence to this indication. The aim of this project (Predict & Prevent) was to evaluate if increase the percentage of patients who started treatment with bisphosphonates/denosumab reduces the bone fracture rates.

## 2. Materials and Methods

A new bone health management model was developed in Italian cancer centers, distributed in the three geographical macroareas (North, Central, South and Islands). Each breast center must treat a minimum of 150 new cases per year, as stated by EUSOMA [35].

The Predict & Prevent project included a new bone health management model, including the following:

1. Training of breast multidisciplinary teams and bone health specialists;
2. Presentation and implementation of a bone health management model in every cancer center;
3. Assessment at the start and 12 months after the implementation of the project, of two key performance indicators (KPIs);
4. Assessment of the rates of bone fractures after five years from implementation of this project in every cancer center.

**Figure 1:**
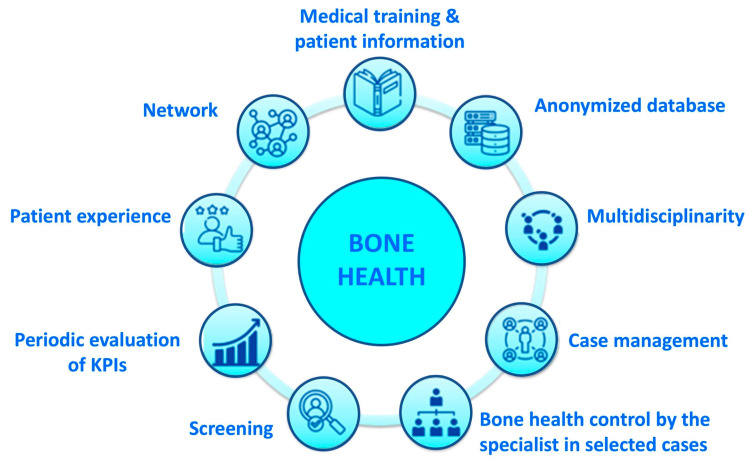
The pillars of the bone health management model: Predict & Prevent.

The pillars of the bone health management model are shown in Figure 1.

### 2.1. Training of Multidisciplinary Team

In 2020, training meetings were organized in every cancer center involved in the project, for members of the breast multidisciplinary group and the rheumatologists/endocrinologists involved in the bone health journey. Due to the COVID-19 pandemic, meetings were virtual. These meetings were coordinated by a single bone specialist and, in each cancer center, by the coordinator of the multidisciplinary breast team of the hospital.

### 2.2. Presentation and Implementation of a Bone Health Management Model

**Figure 2:**
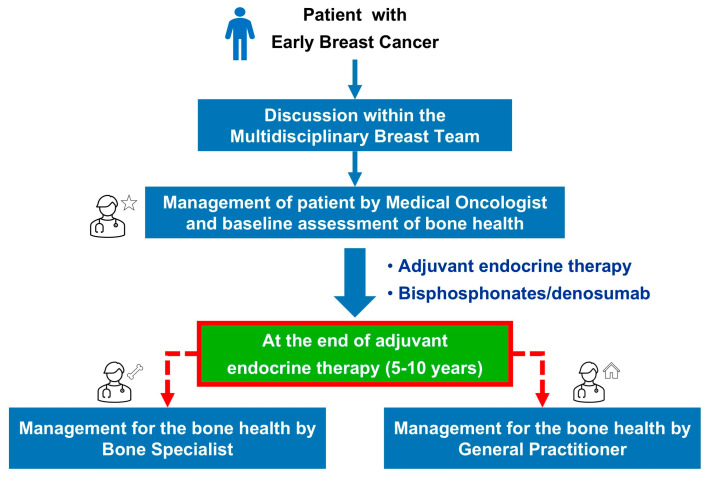
Bone health management model: Predict & Prevent.

After the training meeting carried out in each cancer center, different models of bone health management were evaluated by the various participants in the Predict & Prevent project. The model in which the medical oncologist was the point person for each patient’s bone health was chosen (Figure 2).

Basal bone health evaluation with dual-energy X-ray absorptiometry (DXA), plasma dosage of parathormone (PTH), calcium, and vitamin D.

In the multidisciplinary breast cancer team, every patient with HR+ EBC is evaluated for adjuvant systemic cancer treatment, including endocrine therapy. Criteria of inclusion in this project were as follows: age ≥ 18 years; confirmed diagnosis of early breast cancer; postmenopausal status (natural, surgical, secondary to chemotherapy or GnRH or radiotherapy); indication to adjuvant endocrine therapy (Tamoxifen+GnRH; AI+GnRH; AI); no prior invasive cancer in the previous 5 years. Criteria of exclusion were as follows: contraindication to adjuvant endocrine therapy; patient’s refusal to start adjuvant endocrine therapy; patient’s refusal to undergo health assessment.

In eligible post-menopausal HR+ EBC patients, during the outpatient visit the medical oncologist prescribes the adjuvant ET and initiates the bone health assessment process by requesting dual-energy X-ray absorptiometry (DXA) and plasma dosage of parathormone (PTH), calcium, and vitamin D.

During the subsequent outpatient visit, the medical oncologist evaluates the test results and prescribes bisphosphonates or denosumab according to the indication in AIFA Note 79: zoledronic acid (5 mg intravenously every 12 months), alendronate (70 mg orally per week ± vitamin D), risedronate (35 mg orally per week), or denosumab (60 mg subcutaneously every 6 months), regardless of T-score values. If osteoporosis is diagnosed at baseline, the oncologist refers the patient directly to the bone health specialist.

After the end of the adjuvant endocrine therapy, the patient will be re-evaluated by the medical oncologist: patients with evidence of osteoporosis on DXA (T score less than −2.5) will be referred to the bone health specialist, while patients with evidence of osteopenia (T score between −1 and −2.5) or normal bone density (T score greater than −1) will be referred to the primary care physician (Figure 2).

### 2.3. Identification and Evaluation of Two Key Performance Indicators (KPIs)

**Figure 3:**
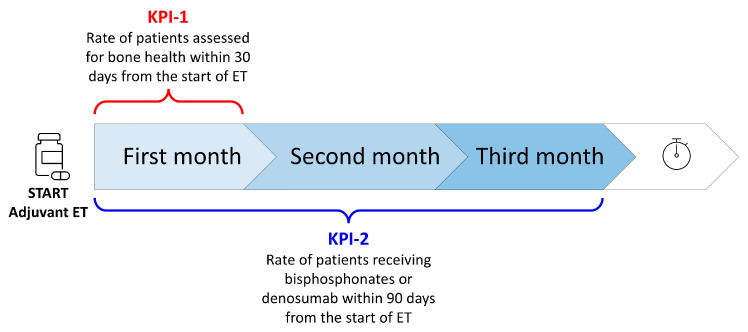
Key performance indicators (ET = endocrine therapy).

Members of the working group of Predict & Prevent project identified two indicators:

- Key performance indicator no. 1 (KPI-1) was “the rate of HR+ breast cancer patients in postmenopausal status (natural, surgical, secondary to chemotherapy or hormonal blockage) assessed for bone health within 30 days from the start of adjuvant endocrine therapy”;
- Key performance indicator no. 2 (KPI-2) was “the rate of HR+ breast cancer patients receiving bisphosphonates or denosumab within 90 days from the start of adjuvant endocrine therapy” (Figure 3).

To evaluate these indicators, each cancer center used available computerized sources of health information from 2020 to 2022. In each hospital, data were anonymized so they could not be traced back to the single patient or the single administrative form.

Each indicator was calculated separately for each cancer center involved in the Predict & Prevent project.

The two key performance indicators were assessed in every cancer center at the following times:

- Time T0 refers to a period of 12 months preceding the start of this project;
- Time T1 refers to a period of 3 months after the start of this project;
- Time T2 refers to a period of 6 months from the start of this project;
- Time T3 refers to a period of 12 months from the start of this project.

The rates of two KPIs at 12 months after the start of the new model (T3) were compared to the rates recorded at time 0 (T0) in each participating cancer center.

### 2.4. Rates of Bone Fractures

The rates of bone fractures will be evaluated after five years from implementation of this project in every cancer center.

### 2.5. Statistical Analysis

The analysis was based on descriptive statistics applied to the results of two key performance indicators (KPIs) reported in every center. Each indicator was calculated as the proportion of patients, among the eligible ones, who received the specified procedure/therapy in the defined time.

Each indicator was calculated separately for each cancer center involved in this project.

Key performance indicator no. 1 (KPI-1) was “the rate of HR+ EBC patients in postmenopausal status (natural, surgical, secondary to chemotherapy or hormonal blockage) assessed for bone health within 30 days from the start of adjuvant ET”. To evaluate this rate, the denominator was represented by all postmenopausal HR+ EBC patients who had started adjuvant ET within 12 months of the start of the Predict & Prevent project in each participating cancer center; the numerator was represented by the number of postmenopausal HR+ EBC patients who had started adjuvant endocrine therapy AND who had been assessed by the oncologist for bone health within 30 days from the start of adjuvant endocrine therapy.

Key performance indicator no. 2 (KPI-2) was “the rate of HR+ EBC patients receiving bisphosphonates or denosumab within 90 days from the start of adjuvant ET, according to the AIFA determination”. To evaluate this indicator, the denominator was represented by all postmenopausal HR+ EBC patients who had started adjuvant ET within 12 months of the start of Predict & Prevent in each participating cancer center; the numerator was represented by the number of postmenopausal HR+ EBC patients who had started adjuvant ET and who had started bisphosphonates or denosumab within 90 days from the start of the adjuvant ET.

A descriptive analysis was applied to the results of both KPI-1 and KPI-2 reported by every cancer center at time T0, time T1, time T2, and time T3 to evaluate the performance of each individual center over time and the impact of the implementation of a new bone health management model on the performance of both KPIs at time T3 versus that at time T0 (primary endpoint of this project).

In an exploratory analysis, the rates of two KPIs reported by the Italian cancer centers involved in this project were evaluated globally at time T0, time T1, time T2, and time T3.

Furthermore, the rates of both KPIs globally reported at time T3 in all cancer centers (12 months after the implementation of this project) were compared to the rates of the KPIs reported by a previous National survey conducted by IQVIA in 2019 to evaluate the impact of this new model on clinical practice.

In this first analysis, we reported the rates of two KPIs 12 months after the start of the new model (T3) and the comparison with the rates recorded at time 0 (T0) in each participating cancer center, to assess whether these percentages had increased after the implementation of the new organizational model.

## 3. Results

**Figure 4:**
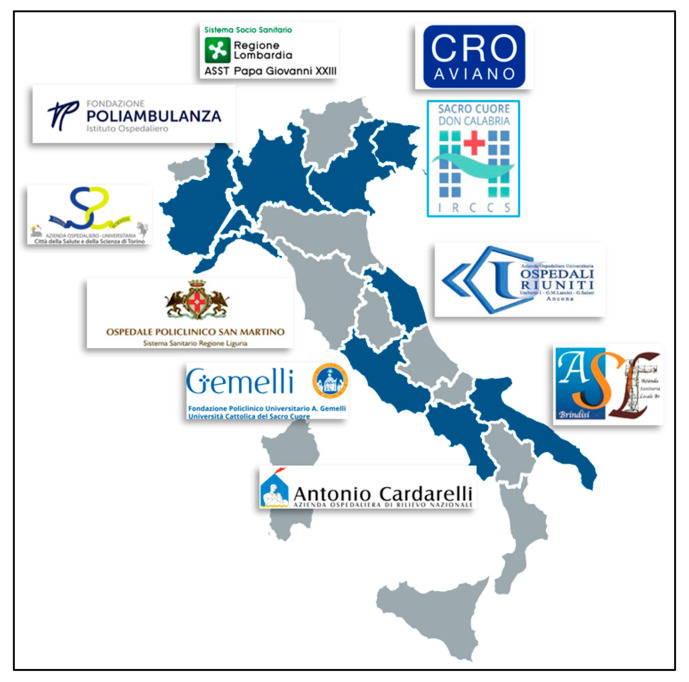
Cancer centers involved in the bone health management project.

From 2020 to 2022, this new bone health management model was developed in 10 Italian cancer centers, distributed in the three geographical macroareas (North, Central, South and Islands) (Figure 4).

This model was implemented in each cancer center at different times between 2021 and 2022. Time T1, time T2, and time T3 refer to different months and years between the different cancer centers due to the different periods of implementation of the new bone health management model.

**Figure 5:**
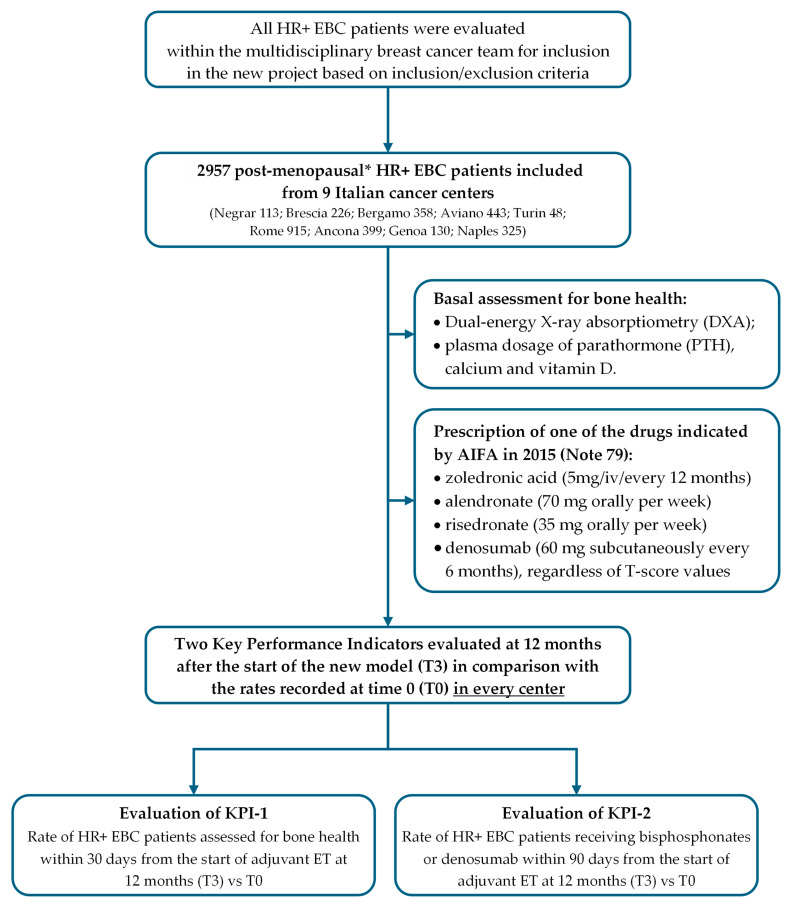
Flow chart of the study and KPIs evaluated in this first analysis. HR+: hormonal receptor-positive; EBC: early breast cancer; ET: endocrine therapy; OFS: ovarian function suppression; AI: antiaromatase inhibitor; KPI: key performance indicator. * Post-menopausal status: natural, surgical, or from chemotherapy or GnRH or radiotherapy.

In one hospital (Ospedale Summa-Perrino, Brindisi), after the training and presentation, the new bone health management model, was not implemented and the two KPIs were not assessed. In September 2023, 9 out of 10 cancer centers involved in this project reported the performance of the two key performance indicators assessed in their hospital (IRCCS Ospedale Sacro Cuore Don Calabria, Negrar; Centro di Riferimento Oncologico IRCCS, Aviano; ASST Papa Giovanni XXIII, Bergamo; Fondazione Poliambulanza, Brescia; Ospedale Molinette, Turin; IRCCS Ospedale Policlinico San Martino, Genoa; Ospedali Riuniti Ancona; Policlinico Gemelli IRCCS, Rome; Azienda Ospedaliera A. Cardarelli, Naples). The anonymized data of a total of 2957 post-menopausal HR+ EBC patients treated with endocrine therapy were included in this evaluation (Figure 5). For every cancer center, the rate of two KPIs at basal time (time T0) and after the implementation of the new management model of bone health (time T1, time T2, and time T3) were reported in Table 1.

The results showed that in each of the six cancer centers (Negrar, Brescia, Bergamo, Aviano, Turin, Rome), the rates relative to KPI-1 (rate of HR+ EBC patients assessed for bone health within 30 days from the start of adjuvant endocrine therapy) and to KPI-2 (rate of HR+ breast cancer patients receiving bisphosphonates/denosumab within 90 days from the start of adjuvant endocrine therapy) increased progressively from time T0 to time T3, due to the training of the multidisciplinary teams and the implementation of the new bone health management model (Table 1). In these six centers, for KPI-1, the range of rates was 0–35% at time T0 and 2–88% at time T3; for KPI-2, the rate range was 0–61% at time T0 and 8–69% at time T3.

In the other three cancer centers (Ancona, Genoa, Naples), where the rate of evaluation of bone health (KPI-1) and the indication to bisphosphonates/denosumab (KPI-2) in HR+ EBC patients was already high at T0, the rates remained high also at T3 (Table 1). In these three centers, for KPI-1, the range of rates was 95–100% at time T0 and 76–100% at time T3; for KPI-2, the rate range was 47–95% at time T0 and 38–98% at time T3.

Some slight fluctuations in the KPIs rates in two out of these three centers were the result of organizational problems. At the oncology center of Ancona (AOU Ospedali Riuniti, Ancona), as a consequence of the reduction in elective surgical activity linked to the COVID-19 pandemic, at time T0 and time T1 relative to KPI-1 a shorter latency in taking charge of patients in comparison with time T2 and time T3 was recorded. In the second cancer center (IRCCS San Martino, Genoa), due to a change in the organization during the data collection period, at time T2 and time T3 of the evaluation of KPI-2, there was a greater delay in the prescription of drugs indicated by AIFA Note 79 than at time T1 and time T0.

**Figure 6:**
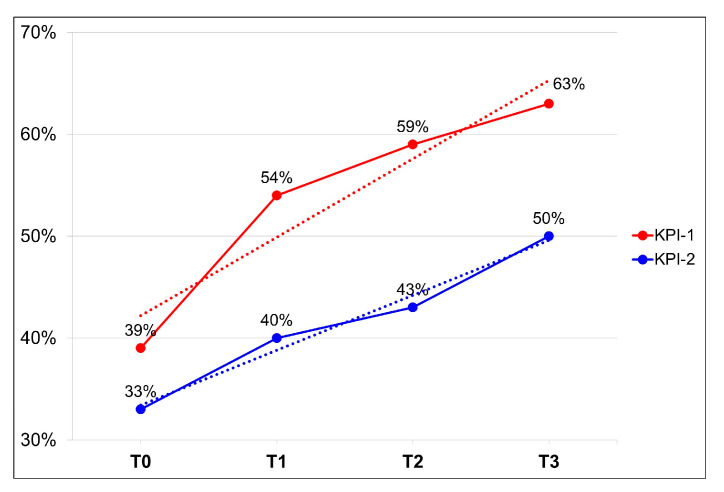
Average values of two KPIs assessed overall, from time T0 to time T3 (T0 refers to a period of 12 months preceding the start of this project; T1 refers to a period of 3 months after the start of this project; T2 refers to a period of 6 months from the start of this project; T3 refers to a period of 12 months from the start of this project). Dotted lines represent the upward trend lines for both KPIs.

In an exploratory analysis, the rates of two KPIs reported by nine cancer centers were evaluated overall (Figure 6). Between time T0 and time T3, an increase in the rate of HR+ EBC patients assessed for bone health within 30 days from the start adjuvant ET (KPI-1) and of the rate of HR+ EBC patients receiving bisphosphonates or denosumab within 90 days from the start adjuvant ET (KPI-2) was observed. In this analysis, the average values for both KPIs show an upward trend over time. The mean for KPI-1 increased from 39% at time T0 to 63% at time T3 (+62%), and for KPI-2 from 33% at time T0 to 50% at time T3 (+52%) (Figure 6).

The most significant improvement for both KPIs was observed between T0 and T1, when KPI-1 registered a 39% increase and KPI-2 registered a 21% increase. This steady increase is largely attributable to centers IRCCS Ospedale Sacro Cuore Don Calabria in Negrar and ASST Papa Giovanni XXIII in Bergamo. Notably, in IRCCS Ospedale Sacro Cuore Don Calabria in Negrar, KPI-1 progressed from 0% to 96% and KPI-2 from 0% to 46%. Similarly, ASST Papa Giovanni XXIII in Bergamo improved KPI-1 from 0% to 98% and KPI-2 from 61% to 98%. Following this initial boost both KPI-1 and KPI-2 kept growing consistently.

A comparison between rates of KPIs reported by a previous National survey conducted in 2019 and rates of KPIs at T3 reported after the implementation of the new model in nine cancer centers, was also performed. This exploratory analysis showed a benefit in the management of bone health after the implementation of the new model. Indeed, in nine cancer centers globally considered, 63% (1870 out of 2957) of HR+ EBC patients were assessed for bone health within 30 days from the start of adjuvant endocrine therapy (KPI-1) compared to 43% (862 out of 1996) observed in the 2019 National survey, and 50% (1473 of 2957) started bisphosphonates/denosumab within 90 days from the start of adjuvant endocrine therapy (KPI-2) in comparison with 25% (506 out of 1996) reported by the survey.

## 4. Discussion

The adjuvant endocrine therapy recommended by international and national Guidelines [6,7] in HR+ early breast tumors induces a marked tissue hypoestrogenism: the consequent accelerated bone loss and impaired bone structure quality result in an increased risk of fractures, which often occur even with normal bone density.

These events are clinically relevant, with a significant impact on morbidity and patients’ quality of life [12,23], increased mortality in the case of hip fractures, and high economic impact on the health care system worldwide [36].

The use of bisphosphonates or denosumab during adjuvant endocrine therapy increases or preserves BMD and reduces the risk of fractures [26,27,28,29,30,31].

In 2015, the Italian Drug Agency (AIFA) published Determination n.589 (Note 79) indicating the use of bisphosphonates (zoledronic acid, alendronate, risedronate) or denosumab for the primary prevention of bone fractures in women with postmenopausal breast cancer undergoing adjuvant ET, regardless of T-score values, with approval and coverage of this treatment by the National Health Service (NHS) [34]. As an Italian survey conducted by IQVIA in 2019 revealed low adherence to the AIFA indication, a new bone health management model was developed in 10 cancer centers between 2020 and 2022. The results of our analysis showed that the implementation of this bone health model increased the adherence to the AIFA indication in six out of nine cancer centers: after the implementation of this model, the rates relative to KPI-1 (rate of HR+ EBC patients assessed for bone health within 30 days from the start of adjuvant ET) and to KPI-2 (rate of HR+ breast cancer patients receiving bisphosphonates or denosumab according within 90 days from the start of adjuvant ET) increased progressively from time T0 to time T3 (12 months from the start of this project).

In the other three cancer centers, where the rate of evaluation of bone health (KPI-1) and the indication to bisphosphonates/denosumab (KPI-2) in HR+ EBC patients was already high at time T0, the rates remained high also at time T3.

These results are very important for the prevention of fragility fractures and quality of life improvement of postmenopausal HR+ EBC patients receiving adjuvant ET, as well as for a reduction in the economic impact of the consequences of bone fractures on the national health care system.

The exploratory analysis of the overall KPIs results reported in all 9 Italian cancer centers showed a better outcome after the implementation of this model in comparison with the data reported from the 2019 survey: 63% of HR+ EBC patients who started adjuvant ET were assessed for bone health within 30 days compared to 43% reported by the previous national survey, and 50% of patients started bisphosphonates/denosumab within 90 days compared to 25% of the survey.

The two most important characteristics of this model are the evaluation of the bone health of every patient with HR+ EBC within the multidisciplinary breast cancer team and the role of the medical oncologist. During the first outpatient visit after the discussion within the multidisciplinary team, the medical oncologist will prescribe both adjuvant endocrine therapy and examinations to assess bone health. Next, within 90 days from the start of adjuvant ET, the medical oncologist will prescribe bisphosphonates or denosumab for the primary prevention of bone fractures in patients with normal BMD or osteopenia and refer patients with osteoporosis at baseline to the bone health specialist. Because every treatment decision and monitoring strategy concerning bone health should involve a collaboration between oncologists, rheumatologists, endocrinologists, and general practitioners [24], this model of bone health management requires various specialists to be included in the team, as needed.

Another important aspect of this model is the involvement of the patient in any other risk factors (smoking, alcohol consumption, sedentary lifestyle, diet, advanced age, steroid use, and family history) for CTIBL and on the toxicity of bisphosphonates and denosumab [37].

This analysis has some limitations. In the first place, adherence to the AIFA Note 79 indication was evaluated only up to 12 months after project implementation and not beyond. Second, the evaluation of the incidence of skeletal fractures before and after the implementation of the new model was not done. However, based on literature data, it can be hypothesized that increasing the percentage of patients treated with adjuvant bisphosphonates or denosumab during ET can reduce the incidence of bone fractures.

In addition, although the BMD is a main predictive factor for fracture and therefore a surrogate endpoint for their prevention in osteoporosis settings, the pathophysiology underlying CTIBL, specifically in EBC patients undergoing adjuvant endocrine therapy, differs from that of primary and postmenopausal osteoporosis, with a prevalent role of bone quality alterations rather than quantity [38]. Further studies are therefore necessary to assess the clinical, molecular and genetic characteristics of patients with EBC, to identify the risk factor of bone loss and to prevent this condition.

Finally, no statistical tests were applied, and the observed differences might have been influenced by organizational factors beyond the intervention. Nevertheless, some barriers to implementation of this new bone health model should be considered (e.g., organizational changes, COVID-19 pandemic impact).

In conclusion, choosing the best organization can lead to potential health benefits for patients with HR+ EBC, and these results showed a model of bone health management that can be implemented in all cancer centers with active multidisciplinary teams.

## 5. Conclusions

The implementation of a new organizational model for bone health management in postmenopausal HR+ EBC patients undergoing adjuvant ET demonstrated a tangible impact on adherence to AIFA recommendations. After 12 months from the implementation of the Predict & Prevent project, six out of nine participating cancer centers reported a significant increase both in the proportion of patients assessed for bone health within 30 days from the start of adjuvant ET (KPI-1) and in the proportion of patients receiving bisphosphonates or denosumab within 90 days (KPI-2). In the remaining three centers, where baseline adherence rates were already high, these results were maintained over time, confirming the robustness of the existing clinical pathways.

These findings highlight the importance of training multidisciplinary teams, engaging bone health specialists, and adopting shared protocols as key strategies to improve clinical management bone health and ensure the application of national guidelines.

This simple but well-coordinated organizational model, led by the medical oncologist and supported by multidisciplinary collaboration, can improve adherence to national bone health guidelines in real-world practice.

The results of this first analysis demonstrate that this new model should be adopted in all cancer centers to allow adequate management of bone health in all postmenopausal HR+ EBC patients undergoing adjuvant ET, with the ultimate goal of reducing the rate of bone fractures in these patients in subsequent years. The fracture outcomes are pending: the rate of bone fractures will be evaluated five years after the implementation of this project at each cancer center.

## Figures and Tables

**Table 1:** Key performance indicators at time T0 and after implementation of the bone health management model (time T1, time T2, time T3) in 9 out of 10 hospitals involved in the Predict & Prevent project.

Cancer Centers		KPI-1				KPI-2			
	Time:	T0	T1	T2	T3	T0	T1	T2	T3
IRCCS Sacro Cuore Don Calabria, Negrar		0%	96%	96%	74%	0%	46%	46%	67%
Fondazione Poliambulanza, Brescia		12%	43%	50%	62%	1%	8%	13%	18%
ASST Papa Giovanni XXIII, Bergamo		0%	98%	90%	88%	61%	98%	67%	69%
Centro Riferimento Oncologico IRCCS, Aviano		0%	4%	4%	2%	3%	8%	10%	8%
AOU Città della Salute e della Scienza, Turin		13%	25%	31%	38%	45%	50%	50%	56%
Policlinico Gemelli IRCCS, Rome		35%	43%	47%	60%	14%	18%	24%	39%
AOU Ospedali Riuniti, Ancona		95%	92%	82%	76%	95%	90%	83%	79%
IRCCS Ospedale Policlinico San Martino, Genoa		100%	100%	100%	100%	47%	40%	33%	38%
Azienda Ospedaliera Cardarelli, Naples		100%	89%	96%	98%	75%	89%	96%	98%
**Total**		**39%**	**54%**	**59%**	**63%**	**33%**	**40%**	**43%**	**50%**

KPI-1: Rate of HR+ EBC patients assessed for bone health within 30 days from the start of adjuvant endocrine therapy. KPI-2: Rate of HR+ EBC patients receiving bisphosphonates or denosumab therapy within 90 days from the start of adjuvant endocrine therapy.

## Data Availability

The authors can confirm that all relevant data are included in the article.
